# Toward a Clinical Decision Support System for Monitoring Therapeutic Antituberculosis Medical Drugs in Tanzania (Project TuberXpert): Protocol for an Algorithm' Development and Implementation

**DOI:** 10.2196/58720

**Published:** 2024-10-21

**Authors:** Yann Thoma, Annie E Cathignol, Yuan J Pétermann, Margaretha L Sariko, Bibie Said, Chantal Csajka, Monia Guidi, Stellah G Mpagama

**Affiliations:** 1 School of Engineering and Management Vaud HES-SO University of Applied Sciences and Arts Western Switzerland Yverdon-les-Bains Switzerland; 2 Centre for Research and Innovation in Clinical Pharmaceutical Sciences Lausanne University Hospital and University of Lausanne Lausanne Switzerland; 3 Kilimanjaro Clinical Research Institute Moshi United Republic of Tanzania; 4 Kibong'oto Infectious Diseases Hospital Sanya Juu United Republic of Tanzania; 5 The Nelson Mandela African Institution of Science and Technology Arusha United Republic of Tanzania; 6 Institute of Pharmaceutical Sciences of Western Switzerland University of Geneva Geneva Switzerland; 7 School of Pharmaceutical Sciences University of Geneva Geneva Switzerland; 8 Service of Clinical Pharmacology Department of Laboratory Medicine and Pathology Lausanne University Hospital and University of Lausanne Lausanne Switzerland

**Keywords:** TDM, therapeutic drug monitoring, pharmacometrics, tuberculosis, model-informed precision dosing software, clinical pharmacology, clinical decision support system, Tanzania, mobile phone

## Abstract

**Background:**

The end tuberculosis (TB) strategy requires a novel patient treatment approach contrary to the one-size-fits-all model. It is well known that each patient’s physiology is different and leads to various rates of drug elimination. Therapeutic drug monitoring (TDM) offers a way to manage drug dosage adaptation but requires trained pharmacologists, which is scarce in resource-limited settings.

**Objective:**

We will develop an automated clinical decision support system (CDSS) to help practitioners with the dosage adaptation of rifampicin, one of the essential medical drugs targeting TB, that is known for large pharmacokinetic variability and frequent suboptimal blood exposure. Such an advanced system will encourage the spread of a dosage-individualization culture, including among practitioners not specialized in pharmacology. Thus, the objectives of this project are to (1) develop the appropriate population pharmacokinetic (popPK) model for rifampicin for Tanzanian patients, (2) optimize the reporting of relevant information to practitioners for drug dosage adjustment, (3) automate the delivery of the report in line with the measurement of drug concentration, and (4) validate and implement the final system in the field.

**Methods:**

A total of 3 teams will combine their efforts to deliver the first automated TDM CDSS for TB. A cross-sectional study will be conducted to define the best way to display information to clinicians. In parallel, a rifampicin popPK model will be developed taking advantage of the published literature, complemented with data provided by existing literature data from the Pan-African Consortium for the Evaluation of Antituberculosis Antibiotics (panACEA), and samples collected within this project. A decision tree will be designed and implemented as a CDSS, and an automated report generation will be developed and validated through selected case studies. Expert pharmacologists will validate the CDSS, and finally, field implementation in Tanzania will occur, coupled with a prospective study to assess clinicians’ adherence to the CDSS recommendations.

**Results:**

The TuberXpert project started in November 2022. In July 2024, the clinical study in Tanzania was completed with the enrollment of 50 patients to gather the required data to build a popPK model for rifampicin, together with a qualitative study defining the report design, as well as the CDSS general architecture definition.

**Conclusions:**

At the end of the TuberXpert project, Tanzania will possess a new tool to help the practitioners with the adaptation of drug dosage targeting complicated TB cases (TB or HIV, TB or diabetes mellitus, and TB or malnutrition). This automated system will be validated and used in the field and will be proposed to other countries affected by endemic TB. In addition, this approach will serve as proof of concept regarding the feasibility and suitability of CDSS-assisted TDM for further anti-TB drugs in TB-burdened areas deprived of TDM experts, including second-line treatments considered important to monitor.

**International Registered Report Identifier (IRRID):**

DERR1-10.2196/58720

## Introduction

Even though tuberculosis (TB) is curable, it remains one of the leading causes of death from a single bacterial infection worldwide. The World Health Assembly declared the end of TB with a drastic reduction in death and incidence; however, the decrease is at a suboptimal pace, only 11% and 9.2%, contrary to at least 20% and 35% by 2020, respectively [1,2]. Sub-Saharan Africa contributes around 25% of the global TB burden, with a higher death rate than other World Health Organization (WHO) regions at 22% [3]. Significant and commonly coexisting comorbidities in patients with TB, which include HIV coinfection [4], enteric pathogens [5], malnutrition [6], and diabetes mellitus (DM) [7], increase the risk of unfavorable treatment outcomes. This is primarily driven by incomplete adherence and pharmacokinetic variability of first-line antitubercular (anti-TB) drugs, leading to insufficient circulating drug exposure and development of drug-resistant TB or excessive exposure and toxicity, resulting in treatment interruption.

Precision dosage of medicines based on drug concentration monitoring is an essential patient-centered approach for optimizing adherence and efficacy and preventing adverse effects. There are available tools for helping pharmacologists with the dosage adaptation process. However, they are difficult to use for nonspecialists and would benefit from automation. Real clinical decision support systems (CDSS), that have the potential to assist practitioners, not necessarily pharmacologists, in the dosage adaptation of anti-TB drugs in patients with TB, are yet to be available in TB endemic settings. Deployment of such an advanced system will encourage the spread of a dosage-individualization culture among practitioners not specialized in pharmacology while bridging the gap by adapting those novel technologies for optimizing TB care [8].

In that context, as part of the Adaptive Diseases Control Expert Programme in Tanzania, the TuberXpert project aims to develop an automated CDSS to help practitioners with the dosage adaptation of rifampicin. Being one of the essential medical drugs targeting TB, rifampicin is known for its significant pharmacokinetic variability and frequent suboptimal blood exposure [9].

This project shows the field application of such an automated CDSS for the dosage adaptation of rifampicin in Tanzania with underpinned applied research questions to address scientific implementation for sustainability. The 3 project partners are the University of Applied Sciences and Arts Western Switzerland (HEIG-VD), the University Hospital of Lausanne (CHUV), and the Kibong’oto Infectious Disease Hospital (KIDH). HEIG-VD is bringing computer science expertise, CHUV pharmacometrics expertise, and KIDH clinical expertise.

## Methods

### Overview

**Figure 1:**
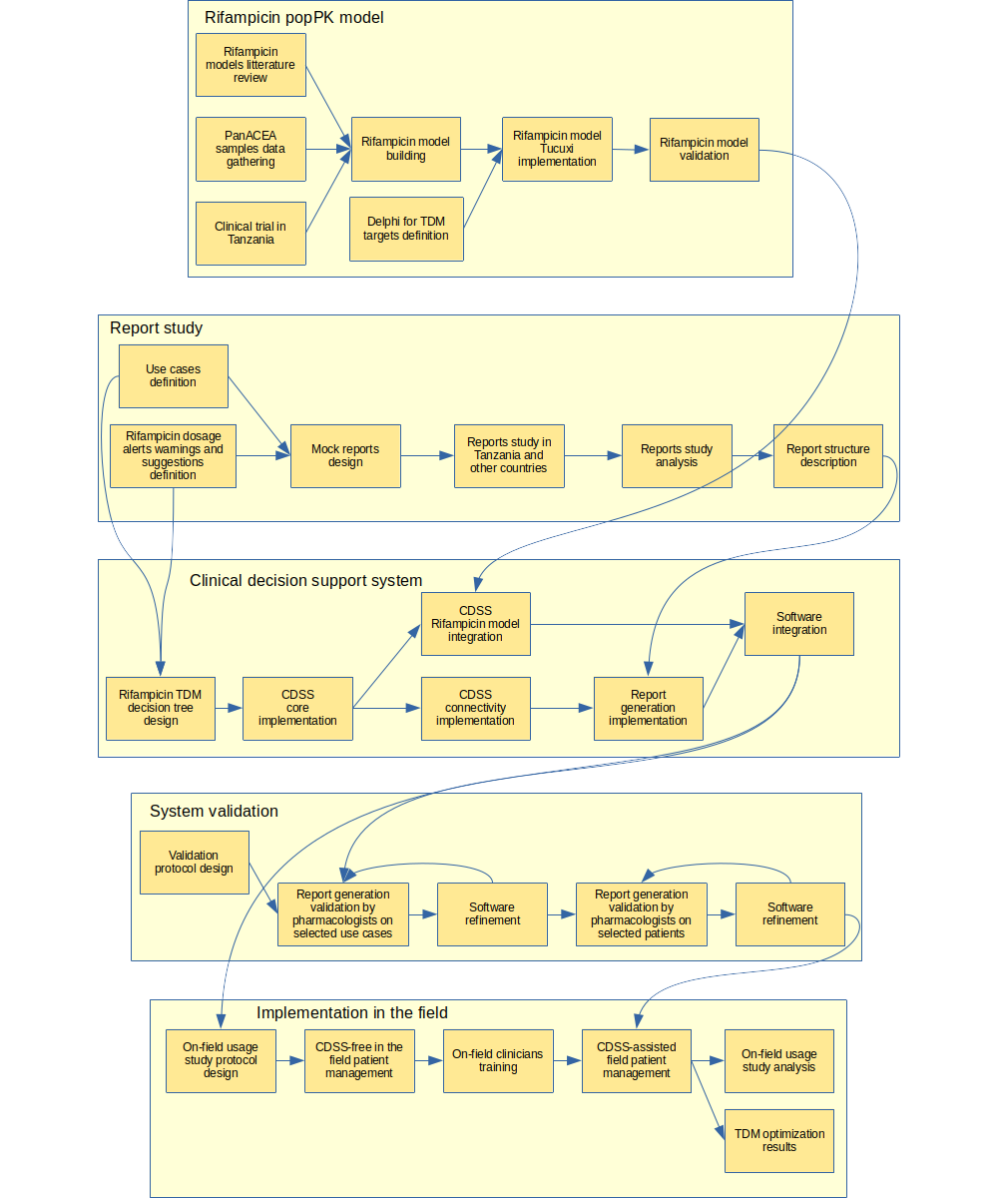
TuberXpert protocol PERT diagram decomposed into 5 phases. CDSS: clinical decision support system; popPK: population pharmacokinetic; panACEA: Pan-African Consortium for the Evaluation of Antituberculosis Antibiotics; PERT: program evaluation and review technique; TDM: therapeutic drug monitoring.

An automated CDSS software requires an embedded population pharmacokinetic (popPK) model for rifampicin to predict drug concentrations and propose meaningful adjustments correctly. The software will be based on the core computing engine of Tucuxi [10,11], a model-informed precision dosing software for Bayesian forecasting that was already developed and that helps clinicians adapt medical drug dosages. These software computations are based on the popPK models implemented within the software and the model-identified influential patient covariates, potential drug concentration measurements, and specific drug pharmacokinetics (PK) targets validated by therapeutic drug monitoring (TDM) experts for dose optimization. The CDSS will be implemented in 5 phases, as shown in Figure 1. In brief, the first phase includes developing an appropriate popPK model for rifampicin for Tanzanian patients and implementing it within Tucuxi. The second phase will optimize reporting relevant information to practitioners for drug dosage adjustment. The third phase will automate the delivery of the report in line with the measurement of the drug concentration [12]. The fourth phase will validate the system, and the final step will implement the system in the field. Details of each stage and associated research questions are summarized in the next subsections.

### Model Selection, Development, and Calibration

Predicting the concentration of a medical drug in blood for dosage adjustment using Tucuxi crucially requires the selection of a popPK model, as the latter determines the ability of the software to propose the correct dosage regimen for each patient. An extensive literature search will be conducted to identify the published popPK models of rifampicin in patients with TB [13-15], developed using NONMEM (ICON) [16] or Monolix (Lixoft) [17], both widely used computer programs for parametric popPK analyses. Models describing rifampicin PK in patients with TB with the previously mentioned comorbidities, that is, DM, malnutrition, and HIV, will be included to investigate their impact on the anti-TB drug PK. The number of participants in the studies, as well as that of available observations distributed over time, will be considered. The description of the model development steps (eg, structural and statistical considerations, and inclusion of covariates) will be carefully reviewed, and the study results will be judged using standard procedures in population analysis (eg, diagnostic goodness-of-fit plots and type of validation) to ensure the quality of the model and the completeness of the information provided. The available models will be ranked according to a cumulative score based on the previous elements, and the best one will be selected for implementation. Because of the autoinduction and the nonlinear processes underlying rifampicin absorption and elimination, we expect a complex nonlinear popPK model to characterize its PK best (which will require software updates to encompass specific kinetics). If the best-identified model fails to capture essential PK characteristics of rifampicin, the opportunity to use a model-based meta-analysis approach will be investigated to build a comprehensive popPK model using all available pertinent literature. Model refinement will be attempted if the selected model proves to be computationally prohibitive for its implementation in Tucuxi, verifying that the simplified model’s prediction for the targeted exposures is comparable to the original ones. Whatever the choice made for this step, the model will undergo formal validation and adjustment to Tanzanian patients using rifampicin concentrations available through the Pan-African Consortium for the Evaluation of Antituberculosis Antibiotics (panACEA) network and those collected in Tanzania within this project. To achieve this aim, a prospective study will be conducted on 50 patients with TB (including those with HIV coinfection, coexistent DM, or malnutrition) followed at the KIDH or in neighborhood clinics during the early stage of the TuberXpert project. These patients will provide 3 samples randomly on 2 separate occasions, that is, at the treatment start and approximately after 1 week of treatment. We will elaborate and then strictly follow a study protocol. We will also conduct a study comparing dried blood spot analysis [12] to HPLC-UV laboratory analysis [18] on these samples. Finally, we will conduct a Delphi study, as suggested in reference [19], to define the optimal therapeutic target, as this is critical for therapy individualization and there is currently no definitive consensus for rifampicin (various propositions [13,20-22]).

Before using Tucuxi in therapy individualization, we have to conduct further essential preliminary validation step. It will consist of verifying that the dosage regimen propositions made by the software are in line with those made by experienced pharmacologists to perform TDM in their clinical practice. Pharmacologists of the service of clinical pharmacology of CHUV and clinicians at KIDH will be in charge of such clinical validation of the software using selected patients of this study. Data on rifampicin dosage history and concentrations, administration, and sampling times, and all necessary clinical information on the selected patients will be collected. Experienced pharmacologists will choose the most appropriate dosage regimen to achieve the target according to the patient clinical status among those proposed by the software using the graphical user interface (GUI) of Tucuxi. The correctness of the forecasted dosing schedule will be validated against the dosage recommendations made by the TDM experts at CHUV and clinicians at KIDH. After this vital validation step, the model will be made available within the standalone Tucuxi software, allowing users to exploit this new model.

The research question components are as follows: (1) What is the appropriate popPK model that includes the structural and statistical considerations, as well as the influential individual factors, to be incorporated in Tucuxi for optimizing rifampicin dosage in patients with TB in Tanzania? (2) What is the Tucuxi-guided rifampicin dosage adjustment accuracy for anti-TB therapy individualization against experienced pharmacologists in the TDM clinical practice?

### Report Design Specifications

Report generation, specifically the report structure and visual content, is essential to help clinicians with decision-making. It shall be easily understandable by inexperienced TDM pharmacologists. The displayed information must be carefully selected and structured to not lose the prescriber with a too complex presentation and terminology [23]. It should contain the appropriate information presented visually. In addition, alerts shall be issued, if necessary, in a form following the Instrument for Evaluating Human Factors Principles in Medication-Related Decision Support Alerts (I-MeDeSa) principles [24].

The definition of the report layout will be elaborated through the following process. We will prepare mock reports showing various layouts based on use cases. The designs will be done after reviewing the literature to identify good practices. We will then, in Tanzania, expose a cohort of clinicians to these reports and collect their input through semistructured interviews. We expect to reach 10 clinicians from different facilities, in Tanzania. We also aim to ask 10 non-Tanzanian clinicians to obtain helpful information about varying preferences regarding information representation depending on culture or background. Thus, pharmacologists with strong expertise in dosage adjustment will be included to get their specific needs compared to general medical doctors. We will improve the report’s rendering over 2 cycles of interviews, with thorough attention to the clinicians’ feedback. The output of this study will allow for the design of the final report. This study will also illustrate the differences between TDM expert pharmacologists and general medical doctors and, hopefully, between other countries and Tanzania for report design acceptance.

The research question components are as follows: (1) What information and how this information shall be displayed to clinicians for an efficient decision-making process concerning dosing regimen? (2) What are the differences in terms of expectations about reports data and design among expert pharmacologists and medical doctors, both in Tanzania and the Western world?

### CDSS

Tucuxi offers a GUI, graphs with drug concentration predictions surrounded by percentiles, and dosage adaptation propositions. The software uses Bayesian inference to find the most likely individual parameters integrating information from the underlying popPK model and the observations obtained on the patient. Currently, the user has to decide by himself or herself on the dosage adjustment among those proposed by the software and write some sentences to fill up a report. Experienced pharmacologists do this manually, but this practice requires particular skills related to this specialized medical discipline. Clinicians who make final decisions about the drug dosage rely on monographs, experience, or a consultation with a pharmacologist for dosage adaptation. In that context, a CDSS can bring much-needed information to decision makers [25] in locations where expert pharmacologists are not readily available.

The CDSS that will be developed in TuberXpert will offer various features and they are (1) assess the expectedness (likelihood) of a drug concentration result, taking into account the patient’s characteristics; (2) assess the adequateness (target attainment) of the current dosage; (3) propose a dosage adjustment if required; (4) present clear and meaningful messages within the report to help the clinician with the decision-making process; and (5) generate alerts when data seem suspicious or erroneous.

While the current Tucuxi software allows the necessary computations, this project will add a new level to the computing engine. Research is needed to ensure the system is extensible enough to address other medical drugs without colossal refactoring. Therefore, a perfect balance between genericity and rifampicin-specific features has to be found. The system must also be easily integrated into existing IT systems thanks to a generic interface. It will also need to be highly reliable and deployable in the field.

Common and uncommon use cases (eg, type of patient, under or over drug exposure, and standard or strange observed drug level) will be identified in both Switzerland and Tanzania, to increase case heterogeneity. Indeed, the patients with TB treated in Switzerland are usually exceptional cases compared to the daily routine patients with TB in Tanzania. Artificial use cases will complete this panel to cover as many borderline cases as possible. We will carefully analyze every patient and use case scenario to design a formal decision tree [26] that will lead to software development. This decision tree will be required to create the software and will be published as a guide for clinicians doing the job by hand. Special attention will be given to appropriate corrective actions [27]. For instance, if a measured drug level appears out of range, an alert shall identify either a potential error in the data or a lack of adherence whenever such sources of bias can be identified with sufficient certainty. In less clear situations, an estimation of the likelihood of common artifacts versus a definite individual PK alteration in the patient should be issued.

The report to be generated shall display proper values and graphs and offer readable sentences. In close relation with the decision tree, a set of standard customizable sentences will have to be defined. They will then be integrated to offer medically meaningful reports as if a human professional had written them. These sentences will be related to TB and rifampicin. Still, the software will be developed keeping in mind that it should be straightforward to define new sentences for other diseases and medicinal drugs. Thanks to established configurations, the core components will be generic, and a parametrization will enable repurposing as efficiently as possible.

We will conduct a short survey in Tanzania to address the availability of computer infrastructures, printers, and connectivity in clinics and at the doctor’s desk. We also will ensure that the system can be connected to the laboratory information system that manages the transmission of drug concentration measurement results. In addition, having a version of the CDSS where computations are running remotely could be an option if the clinicians in the field do not own computers with enough computing power. If this approach is required, the patient’s data could stay on the clinician’s computer to ensure data privacy.

The research question components are as follows: (1) What data processing leads to the most accurate suggestions based on the available data of a specific patient? (2) What setup is most appropriate for rural clinics where access to computer infrastructures is not ideal?

### System Validation

TuberXpert will then be validated after the CDSS and report generation are completed, considering the rifampicin popPK model has already been validated and calibrated to the target population. We will assess the correctness of the generated reports based on use cases designed within the project among patients on rifampicin followed at CHUV and on the data gathered in Tanzania (retrospective data of patients from CHUV and 25 from Tanzania, ie, randomly selected 50% among hospital or study patients). CHUV patients necessitating a TDM intervention often present an overly complex clinical situation, allowing for report-generation testing in extraordinary cases. Experienced pharmacologists will generate a manual report using the GUI of Tucuxi to individualize the therapy of the selected patients. Such a “standard” document will contain all the essential information to justify the chosen dosage adjustment, including the patient clinical situation, data nonreliability, or inconsistencies. It will serve as the gold standard for the TuberXpert report, which will be compared to it to identify any discrepancies. We will verify the correctness of the provided guidance and the presence of all the necessary clinical information, justifications, and warnings. This step will also allow for the refinement of the report to guarantee the reliability of the information for the final user, that is, a clinician without strong TDM expertise. Before conducting this project phase, we will elaborate a detailed protocol to ensure the most reliable validation.

After this first validation step, we will update the CDSS and the report generation based on the study results and specifically on the feedback of the pharmacologists. Indeed, the CDSS development will be driven by the use cases defined in the project beginning, while the validation will be conducted on another set of retrospective data. As such, some adjustments will likely be required.

After the software update, we will conduct a second validation, following the same protocol but on another set of patients (25 from Tanzania and 15 from CHUV). This second step should end with very few differences between the pharmacologists’ outputs and the generated reports, and we only expect minor software modifications to be necessary after that. Finally, we will again run TuberXpert against all the use cases to validate it against the pharmacologists’ choices made during the 2 validation steps. The research question component is—is the CDSS developed reliable enough in comparison with a human pharmacologist?

### Implementation in the Field

The last project phase consists of the CDSS implementation in the field and a prospective study following a strict protocol that will be elaborated according to Tanzania laws on human research. The goal will be to observe the relevance of the generated reports for the clinicians in Tanzania. KIDH will select a cohort of clinicians actively involved in TB treatment with rifampicin that will identify patients to enroll in the study. This step will assess the benefit of CDSS use versus standard practice in Tanzania. Thanks to the existing network of KIDH, we expect to have at least 30 patients in the cohort treated in 10 facilities in the field.

Before introducing TuberXpert to the clinicians, the selected panel will follow their patients according to standard clinical management while collecting all the needed information for a TDM procedure using a dedicated form that will be elaborated according to CDSS specificities. In addition, the possible dosage adjustments, the patient’s health status evolution (ie, clinical features and pragmatic microbiology parameters), and all other relevant data about the patients will be collected. Blood analysis will be implemented and centralized at Kilimanjaro Clinical Research Institute (KCRI) or at KIDH. The KIDH will use this information to feed the CDSS to determine the correspondence between the CDSS output and the clinicians’ decisions. The CDSS will be used in this phase without interfering with the patient’s treatment.

Then, the consortium will train the clinicians in the field about CDSS usage and report interpretation. This training will not only be beneficial for this project but also for spreading the TDM culture throughout Tanzania.

After the training session, the CDSS will be available at the clinics participating in the study. We will deploy the software in facilities where such deployment is feasible, in a setup that still has to be defined, and also set up a direct communication channel with the Swiss team to help the clinicians with any questions regarding software usage or TDM in general. More clinicians will be involved in this second phase, including but not limited to those who participated in the previous non-CDSS phase, as participation in the first phase is not mandatory to be part of the second one. The study protocol will be written during the project and will strongly focus on the clinical relevance of the CDSS and the acceptance of the software tool [28]. The clinicians will be asked to collect the same information as in the first implementation phase, including clinical features during the patients’ follow-up and pragmatic microbiology parameters. Likewise, we will establish the baseline information on the safety of TDM by observing and assessing patients’ clinical evolution between the 2 phases, which will subsequently offer the opportunity to determine the overall benefit of CDSS-assisted versus standard patient management in Tanzania. This second part will also help identify the cases where TuberXpert has influenced the clinicians, why they would not be keen to follow the suggested regimens, and if the outcome of the dosage adjustment has been positive for the patient. If clinicians scarcely follow the CDSS suggestions, we will investigate the barriers and bottlenecks that hinder its implementation to increase the accessibility of benefits of the TuberXpert technology. The analysis will then be performed by comparing the subgroup of clinicians who followed the CDSS recommendations versus those who were reluctant and included in the first phase to assess the CDSS benefits. The research question component is—are clinicians keen to adhere to the suggestions of an automated CDSS?

### Patient and Public Involvement

The development of this protocol aligns with the patient-centered care approach [29]. This project was designed based on the results of a series of TB research studies in Tanzania that showed individual patients with TB differ and may need different treatment strategies and duration due to variations of disease severity and comorbidities [30-34]. Findings from the described project will be shared with organizations involved in TB management for further refinement before subsequently contributing to shaping the agenda of effective integration of communicable and noncommunicable diseases for policy makers.

### Ethical Considerations

The patient enrollment has been approved at the local health research committee serving Kibong’oto Infectious Diseases Hospital and National Health Research Committee with reference KNCHREC003 and NIMR/HQ/R.8a/Vol.IX/2988. Furthermore, the Ministries of Health and Regional Administrative and Local Government Authority have endorsed implementing this protocol. Dissemination will be done mainly through scientific publications for all project parts. Social media will also be used to gain more visibility. Finally, spreading the usage of the system is a goal envisioned at the end of the project.

## Results

The TuberXpert project started in November 2022. During its first phase, and as of July 2024, a total of 50 patients have been enrolled to gather the required data to build a popPK model for rifampicin. In addition, the panACEA consortium agreed to share and already provided individual rifampicin data collected in three different studies (trial numbers NCT01392911, NCT00760149, and NCT01785186), allowing preliminary popPK model development based on these data. A review of TDM practices for TB treatment in Tanzania has also been conducted within the same timeframe. From January to December 2023, we carried out the report design study. We started with a literature review concerning the best way to present information to clinicians. Then we created mock-up reports and conducted a qualitative study with 2 rounds of interviews (13 Tanzanian and 27 non-Tanzanian clinicians) to end up with the final report layout. In September 2023 we started to CDSS general architecture. In July 2024 the first prototype of the CDSS core system was finalized.

## Discussion

### Principal Findings

The TuberXpert project is a multidisciplinary study blending information technology, clinical expertise, and modeling approaches to deploy precision pharmacotherapy of first-line anti-TB medicine through dosage optimization in TB treatment, thanks to an automated CDSS system. To the best of the authors’ knowledge, there is no existing system offering the same functionality. Some TDM software are available, but they require specific skills and do not end up with a fully automated report generation.

After 18 months, interesting information emerged from the project’s first phases. As seen in other low-resource settings, TB in Tanzania is complicated by socioeconomic factors, comorbidities, and a rigid health care system unsuited to patients who are dual-diseased. Significant pharmacokinetic variability of rifampicin between and within subjects further complicates TB management, potentially leading to unfavorable treatment outcomes. Hence, TDM stands as a considerable asset for addressing the limitations of the generalized “one-size-fits-all” approach by tailoring treatment to patient characteristics. While this personalized approach is appealing, the highly complex pharmacokinetic profile of rifampicin significantly complicates straightforward TDM for nonexpert clinicians. Implementation of TDM is further hindered by features exacerbated by low-resource settings, such as logistical barriers, lack of funding, analytical equipment, or trained pharmacologists suited to TDM. If well implemented in the Tanzanian health care landscape, a CDSS coupled with TDM can provide a practical medical tool facilitating personalized medicine in resource-limited settings contributing to alleviating the TB burden.

The report study emphasized the importance of designing accessible and engaging medical reports, particularly in the context of TDM and CDSS. Simplifying and standardizing report formats can improve comprehension and action ability for clinicians, especially those who are nonexperts. As the TuberXpert project aims to create user-friendly TDM reports for TB treatment, it highlights the necessity of clear visuals and appropriate terminology. Interviews with health professionals revealed that simplicity, clear structure, and effective visuals are crucial, though balancing detailed information with simplicity remains challenging. The study also addressed the complexities of multinational collaboration, stressing the need for consistent and understandable communication across diverse health care environments.

### Future Directions

In July 2024 the team will start with the development of the popPK model based on the acquired data and in parallel prepare and validate the CDSS. These phases are expected to last a year. Finally, the implementation of the system in the field will enable the team to assess the adherence of the clinicians to the proposed adjustments and to observe the real impact of the TuberXpert setup. Shall the conclusions be positive, the system could be deployed in other countries, and extended to other medical drugs.

### Strengths and Limitations of This Study

To our knowledge, this is the first study to investigate the application of CDSS technology at varying health care delivery systems levels to guide TDM in patients with TB in TB-endemic settings, especially where pharmacologists are not available. In the future, routine implementation of TDM-CDSS, particularly for rifampicin, a backbone for TB treatment, is expected to transform TB’s clinical management in resource-limited settings. Anti-TB dosage optimization will improve treatment outcomes of patients who would otherwise succumb or develop drug-resistant TB because of suboptimal drug exposure. This will considerably contribute to the end TB strategy, particularly with arduous forms of TB with either HIV coinfection or coexistent DM or malnutrition.

While the project’s expected impacts are important, some limitations could arise. A lack of computer infrastructure in health facilities may prevent the implementation of a centralized system in resource-limited countries. If such computers are not available, if the network is stable enough, we envision using smartphones thanks to a web server offering the TuberXpert service.

### Conclusions

This game-changing transdisciplinary 3-year project combines technology and pharmacometrics to enable appropriate dosages of anti-TB drugs in patients with TB at various levels of the health care system in TB-endemic settings. TuberXpert thus provides an unprecedented opportunity to optimize and individualize the clinical management of patients with TB in resource-limited settings. Moreover, TDM tends to be increasingly recommended, particularly for treating multidrug-resistant TB with second-line chemotherapeutic agents, when achieving effective and safe concentration exposure is of vital importance [35]. In the end, TuberXpert could be adapted beyond rifampicin and holds promise far beyond TB alone, with indirect benefits in other therapeutic areas such as HIV, cancer, and so forth. At the project half-life, it is too early to draw definitive conclusions, but a set of contributions are expected, for the field of dosage adjustment, TDM, and especially for the patients.

### Contribution to the Field

Findings from this project will provide broad knowledge on the feasibility and appropriateness of dosage correction for individuals with suboptimal drug exposure to medicinal drugs critical for the treatment of a life-threatening condition. The project will concretely validate a model-based dosage adjustment of rifampicin in patients with TB. Through publications, it will enhance evidence-based information applicable in different countries.

Routine implementation of the TDM-CDSS particularly for rifampicin, which is a backbone for TB treatment, will transform the clinical management of TB. The current WHO rifampicin dosage of 10 mg/kg was designed rather empirically without prior evidence derived from preclinical or clinical trials [36]. Researchers have documented rifampicin suboptimal drug levels in at least 50% or more of the TB-treated population [30,37]. However, there is limited evidence on the use of technology to address the gaps [8]. This project will also improve the education of medical doctors in Tanzania concerning the dosage adjustment of rifampicin. The expertise will be made available in remote areas where there is a considerable number of patients with TB.

### Contributions to Patient Care, Disease Management, and Public Health

The project will contribute considerably to the end TB strategy by improving patients’ treatment, particularly with arduous forms of TB (either with HIV coinfection or coexistent DM or malnutrition). It might definitely improve treatment outcomes of patients who would otherwise succumb or develop drug-resistant TB as a result of suboptimal drug exposure [22]. A priori dosage recommendations made in this project as a function of specific patient’s characteristics will already limit the use of dangerous subtherapeutic doses in these fragile populations. Furthermore, TDM can decrease the occurrence of adverse drug reactions in patients by favoring appropriate dosages and preventing overdoses [38]. This project challenges the status quo of empirical treatment toward individualization of therapeutics, a patient-centered approach regarded as an important arsenal for the end TB strategy [39].

### Contribution Toward Implementation of TDM-CDSS as an Innovative Technology

The TuberXpert project fosters collaborations with African or European countries that will enable Tanzania to produce high-quality research on pharmacotherapy while strengthening research capacity, and Switzerland will benefit as well on innovation, science, and entrepreneurship. The proof-of-concept will pave the way to a user-friendly TDM-CDSS that has a good chance of proving applicable to other medicinal drugs.

### Contribution Toward the Community of TDM Users

At the end of the project, the source code of the CDSS and report generator will be publicly available, with a license allowing to use it for nonprofit purposes (possibly to use it for helping clinicians, but not to sell a software). If at some stage in the future a company is interested in commercializing a software embedding parts of the existing source code, a specific license with royalties will be considered as an option for further closed-source exploitation. Both the core engine and the GUI of Tucuxi are available in Open Source [40]. The result of TuberXpert will be proposed within this framework to help as many institutions and countries as possible. The vision is to build a community of institutions revolving around TDM, with participants from various countries able to take advantage of each other to improve patient care. This will be of particular interest to African countries that could have access to such tools for free.

## Abbreviations

CDSS: clinical decision support system
CHUV: University Hospital of Lausanne
DM: diabetes mellitus
GUI: graphical user interface
HEIG-VD: University of Applied Sciences Western Switzerland
I-MeDeSa: Instrument for Evaluating Human Factors Principles in Medication-Related Decision Support Alerts
KCRI: Kilimanjaro Clinical Research Institute
KIDH: Kibong’oto Infectious Disease Hospital
panACEA: Pan-African Consortium for the Evaluation of Antituberculosis Antibiotics
PK: pharmacokinetics
popPK: population pharmacokinetic
TB: tuberculosis
TDM: therapeutic drug monitoring
WHO: World Health Organization

## Data Availability

The datasets generated and analyzed during this study are not publicly available due to patients’ data privacy concerns, but are available from the corresponding author on reasonable request. The source code that will be generated during this study will be shared on GitHub as Open Source, allowing anyone to reuse and improve it in the future.
